# Сахарный диабет 1 типа у детей и подростков г. Москвы. Данные Московского сегмента Федерального регистра больных сахарным диабетом 2015 – 2020 гг.

**DOI:** 10.14341/probl12795

**Published:** 2021-12-30

**Authors:** Е. Е. Петряйкина, Д. Н. Лаптев, И. Г. Воронцова, Н. А. Демидов, Ю. А. Ряполова

**Affiliations:** Российская детская клиническая больница; Национальный медицинский исследовательский центр эндокринологии; Российская детская клиническая больница; Городская больница г. Московский Департамента здравоохранения г. Москвы; Детская городская поликлиника №98 Департамента здравоохранения г. Москвы»

**Keywords:** сахарный диабет 1 типа, распространенность СД1, заболеваемость СД1, ретинопатия, нефропатия, нейропатия, кетоацидоз, диабетическая кома, гипогликемическая кома

## Abstract

**ОБОСНОВАНИЕ:**

ОБОСНОВАНИЕ. Терапия сахарного диабета 1 типа (СД1), до настоящего времени является во многом не решенной клинической проблемой. Несмотря на внедрение в клиническую практику современных препаратов инсулина, устройств для его введения, а также непрерывного мониторинга уровня глюкозы, цели терапии зачастую не достигаются. При этом, международная федерация диабета (IDF) отмечает рост распространенности и заболеваемости СД1 у детей и подростков в мире. Федеральный регистр сахарного диабета (ФРСД) – динамически обновляющаяся база данных больных СД, которая позволяет оценить показатели распространенности и заболеваемости, степень достижение целей гликемического контроля, а также частоту развития осложнений СД.

**ЦЕЛЬ:**

ЦЕЛЬ. Анализ эпидемиологических данных СД1 (распространенности, заболеваемости) у детей и подростков (пациенты с рождения до 18 лет) Москвы по данным ФРСД и оценка их динамики, а также динамики достижения целей гликемического контроля и частоты встречаемости осложнений СД1 за 2015-2020 гг.

**МАТЕРИАЛЫ И МЕТОДЫ:**

МАТЕРИАЛЫ И МЕТОДЫ. Объект исследования – выборка из базы данных Московского сегмента ФРСД когорты пациентов с СД1 моложе 18 лет, состоявших на учете за период 01.01.2015-01.01.2021 гг. Эпидемиологические показатели распространенности и заболеваемости рассчитаны на 100 тыс. соответствующего населения.

**РЕЗУЛЬТАТЫ:**

РЕЗУЛЬТАТЫ. численность детей и подростков, страдающих СД1 в Москве на 01.01.2021 г. составила 4024 чел.  (2962 ребенка и 1062 подростка). За период с 2015 – 2020 гг., отмечался рост распространенности СД1 (возможно за счет повышения качества регистрации данных в ФРСД) и снижение заболеваемости как среди детей, так и среди подростков. Также отмечалось снижение уровня НвА1с и доли пациентов с НвАс1 > 8,0% среди детей с СД1. Как среди детей, так и среди подростков с СД1 отмечалось снижение частоты диабетических ком и кетоацидозов с одновременным ростом частоты тяжелых гипогликемий, а также снижение частоты ретинопатии, нефропатии. При этом, частота нейропатии снижалась среди детей и увеличивалась среди подростков.

**ЗАКЛЮЧЕНИЕ:**

ЗАКЛЮЧЕНИЕ. Полученные данные динамического ведения подростков с СД1 являются основанием для рассмотрения вопроса разработки профильной программы их динамического наблюдения, с учетом необходимости психологической и социальной поддержки пациентов и членов их семей.

## Abstract (en)

**BACKGROUND:**

BACKGROUND: Therapy for type 1 diabetes mellitus (T1DM) is still largely an unsolved clinical problem. Despite the introduction into clinical practice of modern insulin preparations, devices for its administration, as well as continuous monitoring of glucose levels, the goals of therapy are often not achieved. At the same time, the International Diabetes Federation (IDF) notes an increase in the prevalence and incidence of T1DM in children and adolescents in the world. The Federal Register of Diabetes Mellitus (FRDM) is a dynamically updated database of patients with diabetes, which allows assessing prevalence and incidence rates, achievement of glycemic control goals and the incidence of diabetes complications.

**AIM:**

AIM: analyze the epidemiological data of T1DM (prevalence, morbidity) in children and adolescents (patients from birth to 18 years of age) in Moscow according to the FRSD data and to assess their dynamics, as well as the dynamics of achieving the goals of glycemic control and the incidence of T1DM complications in 2015-2020.

**MATERIALS AND METHODS:**

MATERIALS AND METHODS: The object of the study is a sample from the database of the Moscow segment of the FRDM of a cohort of patients with type 1 diabetes under 18 years of age who were registered for the period 01.01.2015-01.01.2021. Epidemiological prevalence and incidence rates are calculated per 100,000 of the relevant population.

**RESULTS:**

RESULTS: the number of children and adolescents with type 1 diabetes in Moscow as of 01.01.2021 was 4024 people (2962 children and 1062 adolescents). Over the period from 2015 to 2020, there was an increase in the prevalence of T1DM (possibly due to an increase in the quality of data registration in the FRSD) and a decrease in the incidence of both children and adolescents. There was also a decrease in the level of HbA1c and the proportion of patients with HbAc1> 8.0% among children with T1DM. Both children and adolescents with T1DM showed a decrease in the incidence of diabetic coma and ketoacidosis with a simultaneous increase in the incidence of severe hypoglycemia, as well as a decrease in the incidence of retinopathy and nephropathy. However, the incidence of neuropathy decreased among children and increased among adolescents.

**CONCLUSION:**

CONCLUSION: The data obtained on the dynamic management of adolescents with T1DM are the basis for considering the development of a profile program for their dynamic observation, taking into account the need for psychological and social support for patients and their families.

## ОБОСНОВАНИЕ

СД 1 типа (СД1) — аутоиммунное заболевание у генетически предрасположенных лиц, при котором хронически протекающий лимфоцитарный инсулит приводит к опосредованной Т-клетками деструкции β-клеток с последующим развитием абсолютной инсулиновой недостаточности, со склонностью к развитию диабетического кетоацидоза (ДКА) [1].

СД1 характеризуется хронической иммуноопосредованной деструкцией β-клеток островков поджелудочной железы, которая приводит в большинстве случаев к абсолютному дефициту инсулина. Разрушение β-клеток происходит с различной скоростью и становится клинически значимым при разрушении примерно 90% β-клеток. СД1 является многофакторным заболеванием, однако конкретные механизмы взаимодействия генетической предрасположенности, факторов окружающей среды, состояния иммунной системы, лежащие в основе СД1, остаются неясными [1].

Заболеваемость и распространенность СД1 у детей и подростков в мире, по данным отчета Международной федерации диабета (IDF, 2019 г.), растет с каждым годом, особенно среди лиц в возрасте до 15 лет. Общий годовой прирост больных СД1 за 2019 г. составил около 3%. В общей сложности 1,1 млн детей и подростков в возрасте до 20 лет в мире страдают СД1. Ежегодно около 98 тыс. новых случает СД1 диагностируется у детей и подростков в возрасте до 15 лет. Это число увеличивается до 129 тыс., когда возрастной диапазон расширяется до 20 лет [2].

Заболеваемость СД значительно варьирует в различных странах:

-самые высокие показатели (более 20 на 100 тыс. детского населения в год) отмечены в Скандинавских странах (Финляндия, Швеция, Норвегия) и Сардинии (Италия);

-к странам с наименьшим риском заболеваемости (менее 3 на 100 тыс. в год) отнесены Чили, Мексика, Китай и др. [1].

Основными медико-социальными проблемами, ассоциированными с СД1, возникающим в детском и подростковом возрасте, являются снижение продолжительности жизни пациентов и высокая частота развития тяжелых, инвалидизирующих осложнений.

Общая численность пациентов с СД1 до 18 лет в Российской Федерации на 31.12.2016 составила 31 727 чел. Распространенность СД1 в 2013–2016 гг. у детей составила 81,0–91,4/100 тыс. детского населения, у подростков — 212,8–209,5/100 тыс. подросткового населения. Заболеваемость СД1 среди детей в 2016 г. составила 14,2/100 тыс. детского населения, подростков — 10,0/100 тыс. подросткового населения [3–8].

С целью снижения медико-социального ущерба, обусловленного глобальным ростом распространенности сахарного диабета, в Российской Федерации (РФ) была разработана Федеральная целевая программа (ФЦП) «Сахарный диабет». Одним из ключевых направлений реализации ФЦП стало создание системы клинико-эпидемиологического мониторинга СД в масштабах всей страны посредством Государственного регистра СД (ГРСД) (на сегодняшний день — Федерального регистра СД (ФРСД)).

Данные регистра позволяют оценить эпидемиологические и клинические показатели СД как в РФ, так и в отдельных регионах и населенных пунктах, а также соответствие реальной клинической практики стандартам ведения пациентов.

ГРСД основан Приказом Минздрава России от 10.12.1996 г. № 404; методологическим и организационным референс-центром регистра стал ФГБУ «Эндокринологический научный центр» (ныне «НМИЦ эндокринологии» Минздрава России).

Организация лечебной и профилактической помощи детям и подросткам с СД1 является одним из приоритетных направлений работы в системе Департамента здравоохранения г. Москвы.

По данным информационной базы Московского сегмента ФРСД, на 01.01.2021 в Москве было зарегистрировано 4024 чел. моложе 18 лет, страдающих СД1.

## ЦЕЛЬ ИССЛЕДОВАНИЯ

Провести анализ эпидемиологических данных СД1 (распространенности, заболеваемости) у детей и подростков (пациенты с рождения до 18 лет) г. Москвы по данным ФРСД и оценить их динамику, а также динамику достижения целей гликемического контроля и частоту встречаемости осложнений СД1 за 2015–2020 гг.

## МАТЕРИАЛЫ И МЕТОДЫ

Место и время проведения исследования

Место проведения. Г. Москва. Проведен анализ базы данных Московского сегмента ФРСД.

Время исследования. Анализ когорты пациентов с СД1 моложе 18 лет из базы данных ФРСД был проведен в период с 15.01.2021 по 01.04.2021.

Изучаемая популяция

Объектом исследования являлась когорта больных СД1 моложе 18 лет, состоявших на учете в детских амбулаторных учреждениях г. Москвы за период с 01.01.2015 по 01.01.2021, сформированная на основе базы данных Московского сегмента ФРСД.

Способ формирования выборки из изучаемой популяции

Из Московского сегмента ФРСД была сформирована сплошная выборка больных СД1 моложе 18 лет, состоявших на учете в детских амбулаторных учреждениях г. Москвы за период с 01.01.2015 по 01.01.2021.

Дизайн исследования

Одномоментный срез показателей выборки больных СД1 моложе 18 лет, состоявших на учете в детских амбулаторных учреждениях г. Москвы за период с 01.01.2015 по 01.01.2021 на основе данных Московского сегмента ФРСД. Срез показателей выполнен на дату: 01.01.2021.

Методы

Критерии включения в исследование:

1.наличие СД1;

2.возраст моложе 18 лет в течение любого временного отрезка за период с 01.01.2015 по 01.01.2021.

Были оценены эпидемиологические показатели: распространенность СД1 на 100 тыс. соответствующего населения, заболеваемость на 100 тыс. соответствующего населения и динамика данных показателей за период 2015–2020 гг.

Кроме того, оценивались показатели достижения целей гликемического контроля по уровню НbА1с: средний уровень НbА1с по возрастным группам, доля пациентов, находящихся на различных уровнях гликемического контроля (<7,0%, 7,0–7,9%, 8,0–8,9%, ≥9,0%), а также динамика данных показателей за период 2015–2020 гг.

Также были оценены частота встречаемости (%) осложнений СД1 (комы, диабетические кетоацидозы, тяжелые гипогликемии, диабетическая полинейропатия, диабетическая нефропатия, диабетическая ретинопатия) и динамика данных показателей за период 2015–2020 гг.

Статистический анализ

Для описания полученных данных были использованы абсолютные значения (число пациентов), средние значения и стандартное отклонение (показатели возраста, длительности заболевания, уровня НbА1с).

Распространенность СД1 и заболеваемость были рассчитаны на 100 тыс. соответствующего по возрастной группе населения.

Частота встречаемости осложнения рассчитывалась как доля пациентов с данным осложнением (%) от общего числа пациентов.

Для статистической обработки данных использовали пакет статистических программ Statistica 10.0.

## РЕЗУЛЬТАТЫ

Общая численность детей и подростков, страдающих CД1, в Москве на 01.01.2021 составила 4024 чел. (2962 ребенка и 1062 подростка).

Распространенность СД1 в Москве c 2015 по 2020 гг. среди детей выросла на 25,4% (с 129,7 до 162,6 на 100 тыс. детского населения) (рис. 1). В 2015–2016 гг. распространенность СД1 среди детей Москвы значительно превышала средние показатели по РФ (89,3 в 2015 г. и 91,4 в 2016 г. на 100 тыс. детского населения) [9].

За период 2015–2017 гг. отмечалось снижение распространенности СД1 среди детей в Москве, вероятно, связанное с процессом перехода на онлайн-версию ФРСД и необходимостью удаления из системы накопившихся за предыдущие годы дублированных записей пациентов. После 2017 г. наметился тренд на повышение данного показателя.

Распространенность СД1 среди подростков г. Москвы в период с 2015 по 2020 гг. также выросла на 30,3% (с 273,6 до 356,5 на 100 тыс. соответствующего населения), что также превышает средние показатели по РФ (201,7 в 2015 г. и 209,5 в 2016 г.) [9] (рис. 2). Среди подростков также отмечается тенденция к небольшому снижению распространенности СД1 за период 2016–2018 гг., после чего формируется восходящий тренд.

Таким образом, динамика распространенности СД1 среди детей и подростков коррелирует между собой и отражает похожие процессы, связанные как с повышением качества регистрации данных за последние 3 года наблюдения, так и с реальным ростом распространенности СД1.

При оценке заболеваемости СД1 среди детей и подростков отмечается снижение данного показателя. Так, заболеваемость среди детей с 2015 по 2020 гг. снизилась на 4,2%, с 32,1 до 28,1 на 100 тыс. соответствующего населения, при этом оставаясь в 2 раза и более выше, чем в среднем по РФ (в РФ в 2015 г. — 15,6, в 2016 г. — 12,4 на 100 тыс. соответствующего населения [9]) (рис. 3). Наибольшая динамика снижения отмечается в период с 2015 по 2016 г., а с 2018 г. наметился рост данного показателя (см. рис. 3).

Заболеваемость среди подростков за период наблюдения снизилась на 13,3%, с 25,5 до 22,2 на 100 тыс. подросткового населения. Данный показатель по Москве также в 2 раза превышает средние данные по РФ (в РФ в 2015 г. — 12,8, в 2016 г. — 10,0 на 100 тыс. соответствующего населения [9]) (рис. 4).

Таким образом, мы одновременно наблюдаем постепенное снижение показателей заболеваемости СД1 среди детей и подростков и тенденцию к росту распространенности данного заболевания. Можно предположить, что данные факты в большей степени говорят о постепенном повышении качества регистрации данных в ФРСД, чем о реальном росте распространенности в данных возрастных группах. Скорее всего, значимую долю пациентов, которые были поставлены на учет в ФРСД за последние 3 года, составляют больные СД1, ранее уже наблюдавшиеся в МО Москвы, но своевременно не зарегистрированные в ФРСД.

При оценке динамики показателей гликемического контроля больных СД1 с 2015 по 2020 гг. можно сделать вывод, что на протяжении всего периода наблюдения уровень НbА1с среди детей был значительно ниже, чем среди подростков. При этом если среди детей отмечается тенденция к снижению данного показателя в течение последних 3 лет наблюдения, то у подростков сложно выделить какой-либо устойчивый тренд (рис. 5).

При анализе динамики распределения по уровню НbА1с у детей отмечается выраженный рост доли пациентов с уровнем НbА1с <7,0%. При этом снижается доля пациентов, имеющих уровень НbА1с >8,0% (рис. 6).

Данные ФРСД по Москве, по сравнению с показателями распределения по уровню НbА1с в РФ за 2015–2016 гг., демонстрируют лучшее достижение целей гликемического контроля. Доля пациентов с уровнем НbА1с >9% в 2015–2016 гг. в среднем по РФ составляла 36 и 35% соответственно [9], в Москве — 21,1 и 21,3% (см. рис. 6).

**Figure:**
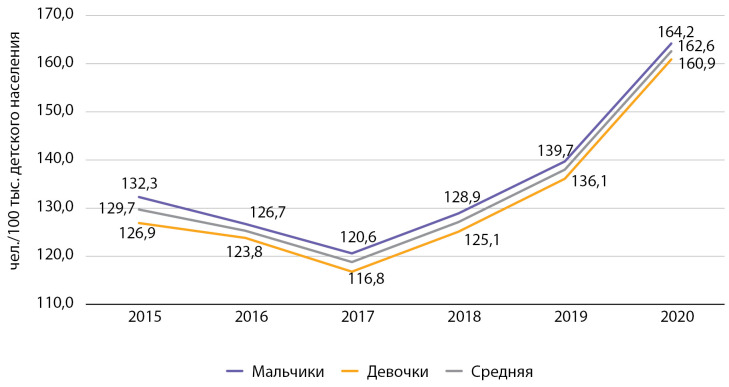
Рисунок 1. Динамика распространенности сахарного диабета 1 типа среди детей с 2015 по 2020 гг. по данным Московского сегмента ФРСД (на 100 тыс. детского населения).

**Figure:**
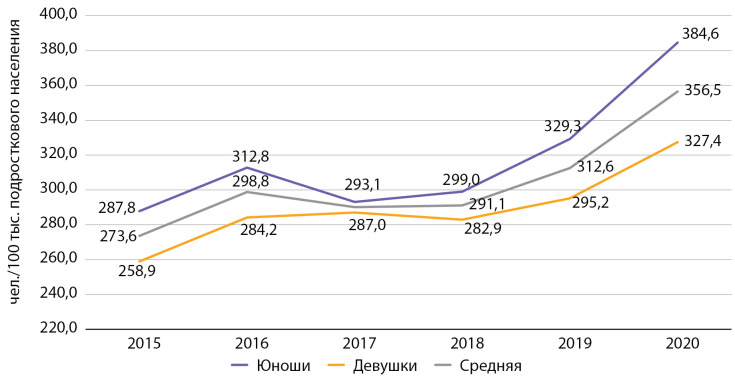
Рисунок 2. Динамика распространенности сахарного диабета 1 типа среди подростков с 2015 по 2020 гг. по данным Московского сегмента ФРСД (на 100 тыс. подросткового населения).

**Figure:**
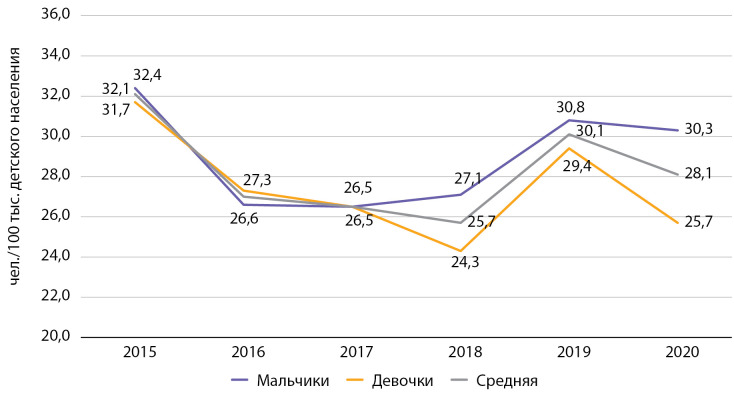
Рисунок 3. Динамика заболеваемости сахарным диабетом 1 типа среди детей с 2015 по 2020 гг. по данным Московского сегмента ФРСД (на 100 тыс. детского населения).

**Figure:**
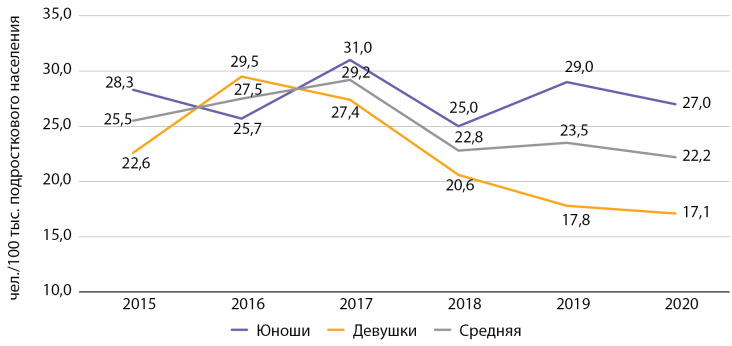
Рисунок 4. Заболеваемость сахарным диабетом 1 типа подростков с 2015 по 2020 гг. по данным Московского сегмента ФРСД (на 100 тыс. подросткового населения).

**Figure:**
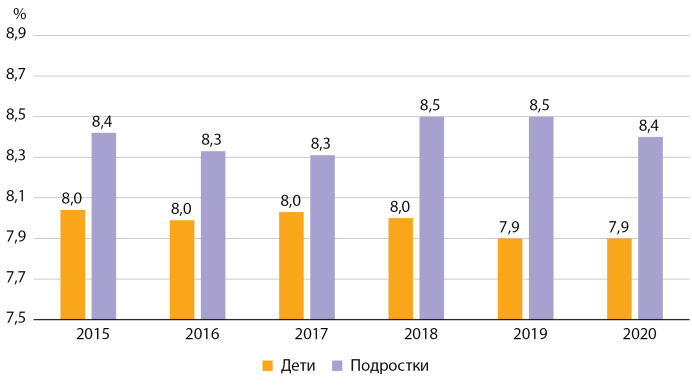
Рисунок 5. Динамика уровня гликированного гемоглобина у детей и подростков с сахарным диабетом 1 типа по данным Московского сегмента ФРСД, 2015–2020 гг., %.

**Figure:**
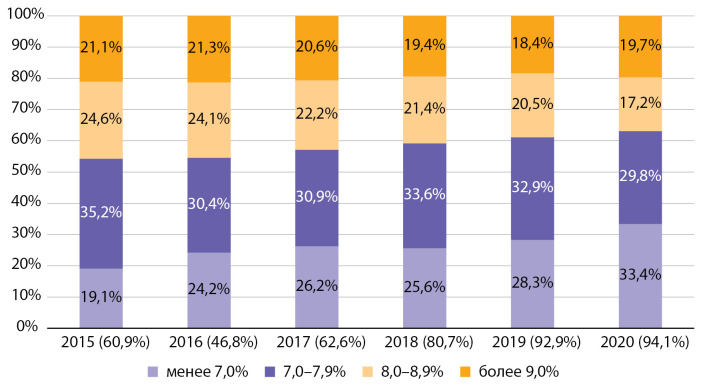
Рисунок 6. Распределение по уровню гликированного гемоглобина среди детей с сахарным диабетом 1 типа по данным ФРСД по Москве с 2015 по 2020 гг. (% числа больных).

При этом за период с 2015 по 2020 гг. отмечается значимое повышение качества обследования на НbА1с. Доля детей с известным уровнем НbА1с выросла за отчетный период с 60,9 до 94,1% (см. pис. 6).

Динамика распределения по уровню НbА1с у подростков не отражает каких-либо устойчивых трендов. В 2015–2017 гг. наблюдался рост доли подростков с уровнем НbА1с <8,0% и снижалась доля пациентов с уровнем НbА1с >8,0%, но с 2018 г. данный тренд сменился на противоположный. В 2020 г. вновь отмечается увеличение доли больных с уровнем НbА1с <8,0% (рис. 7). По сравнению со средними показателями по РФ в Москве среди подростков также отмечается лучшее достижение целей гликемического контроля. Так, в 2015–2016 гг. среди подростков РФ доля пациентов с уровнем НbА1с >9,0% составляла 45% [9], а среди подростков Москвы — 31,0 и 25,5% соответственно (см. pис. 7).

За период с 2015 по 2020 гг. среди подростков, так же как и среди детей, отмечается повышение качества обследования на НbА1с. Доля подростков с известным уровнем НbА1с выросла за отчетный период с 56,3 до 93,6% (см. рис. 7).

Таким образом, на протяжении всего периода наблюдения среди детей с СД1 уровень НbА1с был ниже, чем среди подростков, при этом у детей наблюдается выраженный тренд на рост доли пациентов с уровнем НbА1с <8,0%.

На протяжении периода наблюдения 2015–2016 гг. среди детей и подростков Москвы наблюдались значительно лучшие показатели углеводного обмена, чем средние показатели по РФ при ежегодном повышении охвата обследованием на НbА1с, начиная с 2017 г.

При оценке частоты встречаемости различных осложнений СД1 среди детей и подростков г. Москвы было выявлено, что как среди детей, так и среди подростков наблюдается длительно существующий выраженный тренд на снижение частоты встречаемости комы.

Так, за время наблюдения частота встречаемости комы снизилась в 4,5 раза среди детей и в 2,5 раза среди подростков (рис. 8), что может быть связано с профилактикой развития данного острого осложнения СД1, направленной на улучшение гликемического контроля СД, обучение пациентов и членов их семей. При этом частота встречаемости комы в Москве значительно превышала таковую в среднем по РФ по показателям 2016 г. (в РФ в 2016 г. частота комы составила среди детей 1,2%, среди подростков — 3,2% [9]), что, вероятно, связано с лучшими возможностями диагностики и учета данного состояния в г. Москве.

Частота ДКА за наблюдаемый период также снизилась на 18,5% среди детей и почти в 2 раза — среди подростков (рис. 9). Показатели частоты ДКА в Москве, по данным 2016 г., среди детей в 2,6 раза, а среди подростков в 1,9 раза выше, чем в среднем по РФ (в РФ в 2016 г. среди детей частота ДКА составила 2,5%, среди подростков — 3,2% [9]), что также можно связать с лучшими возможностями своевременной диагностики данного состояния, госпитализации и учета пациентов.

По данным Московского сегмента ФРСД, за период наблюдения отмечается тенденция к росту частоты встречаемости тяжелых гипогликемий. Так, среди детей частота тяжелых гипогликемий за отчетный период выросла в 12,3 раза (с 0,04 до 0,49%), а среди подростков за период с 2017 по 2020 гг. — в 4,2 раза (с 0,2 до 0,8%). При этом важно отметить, что абсолютное число случаев тяжелой гипогликемии в год составляет максимально 15 случаев среди детей (для диспансерной группы 2962 чел.) и 9 случаев среди подростков (для диспансерной группы 1062 чел.). Таким образом, несмотря на выраженный рост данного показателя в процентном отношении, он представлен единичными случаями в абсолютном выражении. Данная ситуация в детском и подростковом возрасте — это, в том числе, неизбежная сторона усилий по достижению целевых показателей самоконтроля, необходимого для профилактики развития инвалидизирующих и смертельных осложнений СД1, особенно при достижении целевого показателя НbА1с менее 7%. Рост доли пациентов с тяжелыми гипогликемиями ни в коей мере не отменяет необходимости достижения целей гликемического контроля, но требует дополнительного обучения пациентов и членов их семьи, применения современных средств мониторинга гликемии.

Частота тяжелых гипогликемий в Москве по данным 2016 г. была значительно ниже таковой в среднем по РФ (в РФ в 2016 г. среди детей 0,3%, среди подростков 0,7% [9]), что может объясняться большими возможностями по обучению пациентов и их родителей, а также высоким уровнем обеспеченности средствами самоконтроля гликемии.

При оценке динамики частоты встречаемости диабетической ретинопатии обращает на себя внимание выраженный тренд на снижение данного показателя как среди детей, так и среди подростков. Так, среди детей данный показатель снизился за период наблюдения в 2,2 раза, среди подростков — в 2,6 раза (рис. 11). По сравнению со средними показателями распространенности данного осложнения в 2016 г. в РФ отмечаются сопоставимые данные (в РФ частота ретинопатии в 2016 г. составляла среди детей 2,7%, среди подростков — 9,8% [9].

За время наблюдения частота диабетической нефропатии среди детей снизилась на 33,4% (с 1,5 до 0,9%), среди подростков — на 47,6% (с 6,2 до 4,2%) (рис. 12). По данным ФРСД по РФ, частота нефропатии в 2016 г. составляла 1,4% среди детей и 8,5% среди подростков [9]. Таким образом, не отмечалось существенных различий по частоте встречаемости данного осложнения среди детей в Москве в среднем по РФ, при этом среди подростков в Москве данное осложнение встречалось в 1,6 раза реже.

При анализе частоты встречаемости диабетической нейропатии обращают на себя внимание тенденция к постоянному росту данного показателя среди подростков (на 37,2% за период наблюдения) и относительно стабильные показатели с тенденцией к снижению за последние 3 года среди детей (на 12,0%) (рис. 13). По сравнению с данными по РФ за 2016 г. можно отметить, что частота нейропатии среди детей в Москве в 2016 г. была в 1,8 раза выше, чем в среднем по РФ (10,9% [9]), а среди подростков сопоставима с данными по РФ (40,5% [9]). Более высокая частота диагностики нейропатии в детском возрасте может быть обусловлена лучшими диагностическими возможностями по ранней диагностике данного осложнения в г. Москве.

**Figure:**
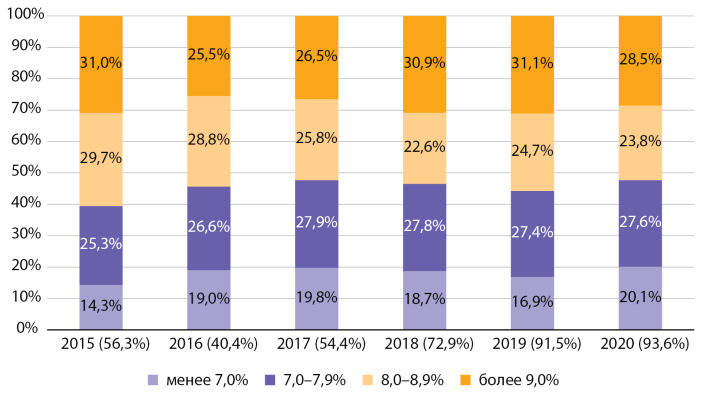
Рисунок 7. Распределение по уровню гликированного гемоглобина среди подростков с сахарным диабетом 1 типа по данным Московского сегмента ФРСД с 2015 по 2020 гг. (% числа больных).

**Figure:**
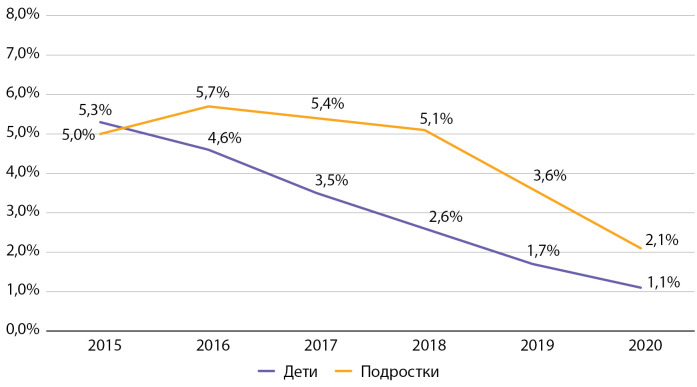
Рисунок 8. Динамика частоты встречаемости состояния комы у детей и подростков с сахарным диабетом 1 типа с 2015 по 2020 гг. по данным Московского сегмента ФРСД (% от числа больных).

**Figure:**
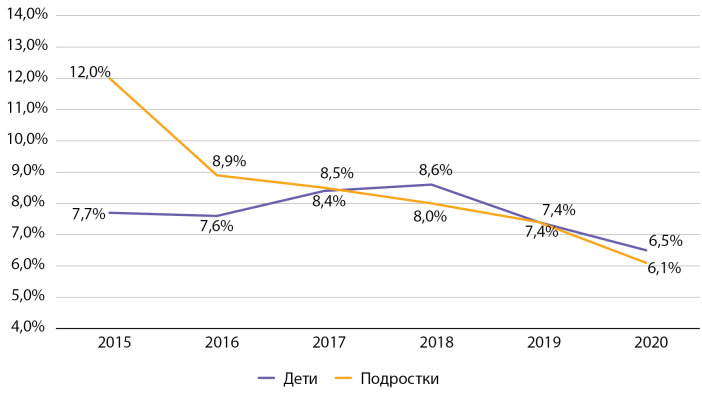
Рисунок 9. Динамика частоты встречаемости диабетического кетоацидоза (без комы) у детей и подростков с сахарным диабетом 1 типа с 2015 по 2020 гг. по данным Московского сегмента ФРСД (% от числа больных).

**Figure:**
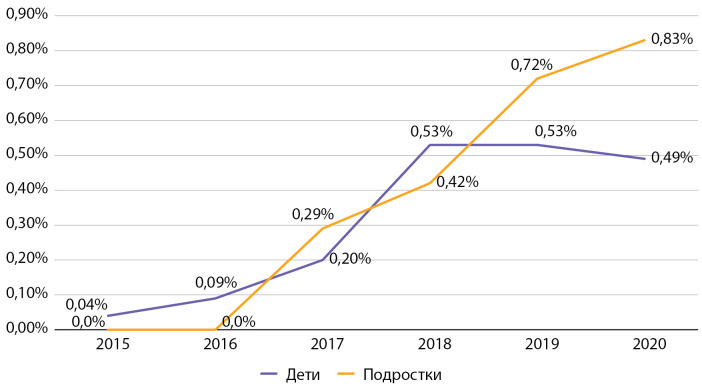
Рисунок 10. Динамика частоты встречаемости тяжелых гипогликемий у детей и подростков с сахарным диабетом 1 типа по данным ФРСД с 2015 по 2020 гг. (% от числа больных).

**Figure:**
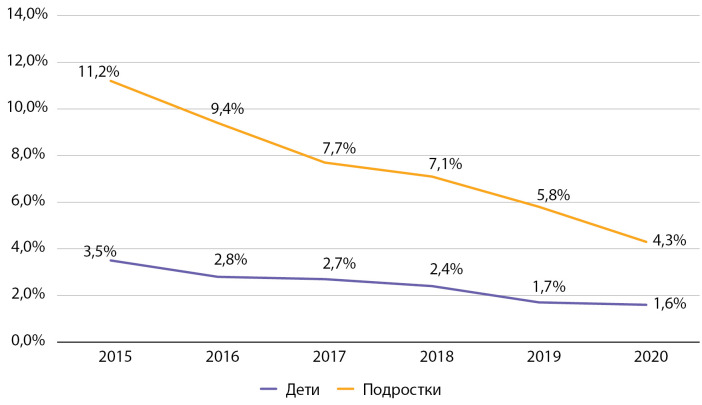
Рисунок 11. Динамика частоты встречаемости ретинопатии у детей и подростков с сахарным диабетом 1 типа по данным Московского сегмента ФРСД с 2015 по 2020 гг. (% диспансерной группы).

**Figure:**
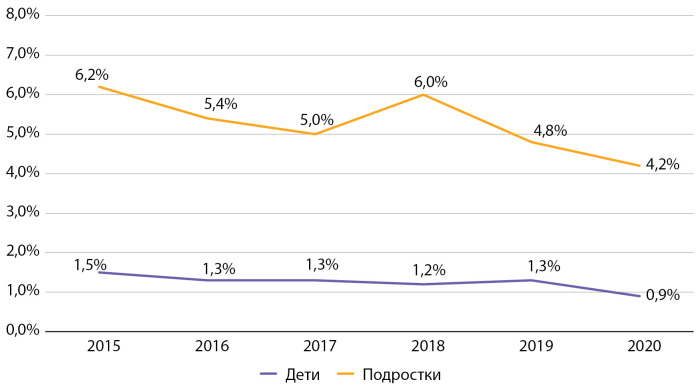
Рисунок 12. Динамика частоты встречаемости нефропатии у детей и подростков с сахарным диабетом 1 типа по данным Московского сегмента ФРСД с 2015 по 2020 гг. (% диспансерной группы).

**Figure:**
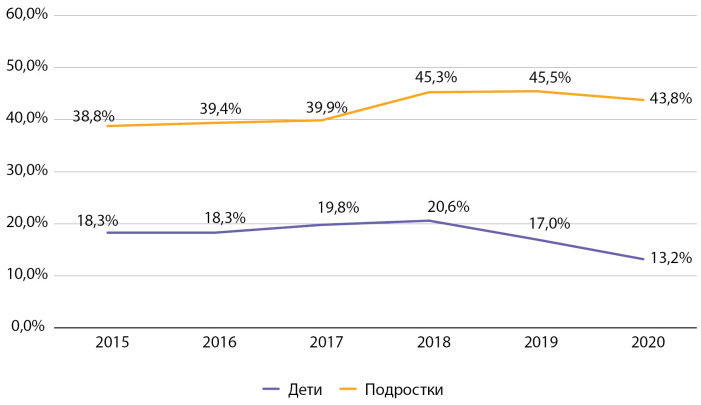
Рисунок 13. Динамика частоты встречаемости нейропатии у детей и подростков с сахарным диабетом 1 типа по данным Московского сегмента ФРСД с 2015 по 2020 гг. (% диспансерной группы).

## ОБСУЖДЕНИЕ

За последние десятилетия произошли глобальные изменения в подходах к терапии СД1. К таким изменениям можно отнести внедрение в клиническую практику аналогов инсулина, инсулиновых помп и мониторинга уровня глюкозы. При этом, хотя и наблюдается рост доли пациентов с уровнем НbА1с <8,0%, но только среди детей. Доля подростков, не достигающих целей терапии, по-прежнему остается высокой.

Тем не менее преимущества внедрения в ежедневную клиническую практику новых препаратов инсулина и современных электронных устройств для введения инсулина и мониторинга уровня глюкозы доказательно продемонстрированы снижением частоты встречаемости диабетических ком и ДКА, а также диабетической ретинопатии, нейропатии и нефропатии (только среди детей). При этом побочным эффектом стремления к достижению более низких цифр гликемии может являться повышение числа тяжелых гипогликемий, хотя общее их количество в абсолютном выражении невелико (в масштабах г. Москвы).

Одновременный процесс снижения частоты встречаемости нейропатии (на фоне улучшения показателей гликемического контроля и снижения частоты ретинопатии и нефропатии) у детей и рост данного показателя среди подростков может говорить как о гипердиагностике данного осложнения, обусловленной в большей степени социальными, нежели медицинскими причинами (стремление сохранить социальные льготы при достижении 18-летнего возраста), так и об инструментальных возможностях доклинического скрининга осложнений СД1 в медицинских организациях Москвы.

Клиническая значимость результатов

Представленные данные демонстрируют положительные результаты медицинского сопровождения детей и подростков в медицинских организациях Москвы. При этом ведение подростков, страдающих СД1, требует дополнительного анализа и обсуждения, возможно, с рассмотрением вопроса разработки профильной программы их динамического наблюдения, с учетом необходимости психологической и социальной поддержки пациентов и членов их семей.

Ограничения исследования

Необходимо отметить, что динамика всех эпидемиологических показателей СД напрямую связана с качеством выявления данной патологии и последующим качеством внесения данных в ФРСД. Уникальная особенность СД1 у детей и подростков в этом отношении заключается в том, что выявляемость данного заболевания составляет практически 100% (в отличие от СД2 у взрослых), поэтому своевременное внесение данных в ФРСД позволяет иметь практически полную картину ситуации с распространенностью и заболеваемостью.

Изложенные выше данные, демонстрирующие высокие темпы прироста распространенности СД1 как среди детей, так и среди подростков, при стабильных или даже снижающихся показателях заболеваемости могут объясняться различным качеством заполнения ФРСД в разные годы. Интенсификация работы по внесению данных в течение последних 3 лет привела к значительному увеличению числа больных в ФРСД и росту показателя «распространенность». Однако это произошло не только за счет введения в ФРСД информации о впервые выявленных больных СД1, формирующих показатель «заболеваемость», но и за счет внесения информации о больных, ранее состоявших на учете в медицинских организациях. Дальнейшее повышение своевременности и качества ввода данных в ФРСД позволит получить максимально приближенную к реальности информацию о динамике распространенности и заболеваемости СД1.

Направления дальнейших исследований

В продолжение данного исследования считаем целесообразным более подробно проанализировать зависимость степени достижения целей гликемического контроля от использования современных устройств для мониторинга уровня глюкозы и непрерывной подкожной инфузии инсулина, приобретающих всю большую распространенность в детской практике терапии СД1.

## ЗАКЛЮЧЕНИЕ

Наша работа демонстрирует текущее состояние медицинского сопровождения детей и подростков в медицинских организациях Москвы и может выступать отправной точкой для формулировки основных направлений дальнейшей организационной работы, направленной на достижение целей гликемического контроля у максимального числа детей и подростков с СД1 г. Москвы, а также на снижение риска развития как острых, так и отдаленных осложнений данного заболевания. Полученные результаты демонстрируют более низкую эффективность проводимых терапевтических мероприятий в когорте подростков с СД1 и предполагают необходимость отдельных мероприятий, направленных на работу именно с этой популяцией больных.

Можно заключить, что ФРСД является эффективным инструментом в комплексной оценке качества оказания медицинской помощи больным СД1. Эффективность внедрения любых комплексных организационных и клинических решений в дальнейшем также может быть достаточно точно оценена с помощью встроенных в ФРСД аналитических алгоритмов. Своевременное внесение клинических данных пациентов в программу ФРСД, а также повышение качества этих данных могут значительно увеличить эффективность оценки проводимых организационных и клинических мероприятий.

## ДОПОЛНИТЕЛЬНАЯ ИНФОРМАЦИЯ

Источники финансирования. Работа выполнена по инициативе авторов без привлечения финансирования.

Конфликт интересов. Авторы декларируют отсутствие явных и потенциальных конфликтов интересов, связанных с содержанием настоящей статьи.

Участие авторов. Все авторы одобрили финальную версию статьи перед публикацией, выразили согласие нести ответственность за все аспекты работы, подразумевающую надлежащее изучение и решение вопросов, связанных с точностью или добросовестностью любой части работы.
